# Characterization of Hepatitis C Virus genotype 3a Hypervariable region 1 in patients achieved rapid virological response to alpha interferon and Ribavirin Combination therapy

**DOI:** 10.1186/1743-422X-8-253

**Published:** 2011-05-23

**Authors:** Madiha Akram, Muhammad Idrees, Abrar Hussain, Samia Afzal, Muhammad Ilyas, Shamail Zafar, Mahwish Aftab, Sadaf Badar, Bushra Khubaib

**Affiliations:** 1 National Centre of Excellence in Molecular Biology, 87-West Canal Bank, Road Thokar Niaz Baig Lahore-53700, University of the Punjab, Lahore, Pakistan; 2 Associate Professor of Medicine, Lahore Medical and Dental College, Lahore, Pakistan

## Abstract

**Background:**

Hepatitis C virus roots a chronic liver disease. Currently approved treatment strategy includes administration of alpha interferon and ribavirin combined therapy for 24-48 weeks. One of the predictor of sustained virological response is an early virological response to treatment characterized as rapid response. Hyper variable region 1 (HVR1) of E2 protein is responsible for viral entry and acts as a target for neutralizing antibodies. Any mutation in this region would effect virus interaction with target cell and viral persistence.

**Methods:**

Thirty one clones of six pre-treatment samples subjected to combination therapy were investigated. Three of the patients were rapid responders (R1, R2 and R3) and two were breakthrough responders (BT1 and BT2). Envelope 2 gene was amplified, cloned and sequenced. Amino acid substitution, frequency, composition and antigenic properties of HVR 1 of E2 protein were studied.

**Results:**

In both rapid responders (R.R) (14 amino acid sites) and breakthrough responders (BT.R) (13 amino acid sites) half of the amino acid sites were either conserved or resistant to any physiochemical change due to amino acid substitution. It also indicated that average composition of hydrophilic and basic amino acids were comparatively lower in rapid responders than other samples affecting probable interaction of virus with target cells. A central non antigenic region was constant among the breakthrough responders but differed in length significantly among rapid responders reflecting the adaptive nature of HVR1 to the immune response.

**Conclusions:**

We observed that although HVR1is quite variable region in HCV 3a patients responding differently to treatment it still maintains its physiochemical properties for its proper functioning and viability.

## Background

HCV roots a persistent infection which advances towards chronic hepatitis, liver steatosis, cirrhosis and hepatocellular carcinoma [1-3]. It is a blood borne infection that infects 270-300 million people worldwide [4]. In Pakistan it affects above 10 million people, almost 8-10% of the total population [5,6].

The standard therapy for HCV treatment adds up to taking Alpha-interferon 3 MU three times a week [7] and 1000-1200 mg of nucleoside analogue ribavirin per day/24-48 weeks [8]. HCV genotype and the viral load figure out the antiviral response and the rate is higher for genotype 2 and 3 than for genotype 1 [9]. Genotype 3a, the most respondent to therapy, which is frequent genotype in Pakistan [10].

In HCV E1 and E2 protein are heterogeneous, especially hyper variable regions which include HVR1, a 27 amino acid sequence (81 nucleotides) [11,12]. Many studies stated that HVR1 lower complexity favours response [13]. Some studies however pointed out negative correlation between genetic heterogeneity and treatment response [14,15]. It has been suggested that HVR1 region of E2 protein act as a target for neutralizing antibodies [16] and is involved in viral entry [17,18]. Rapid changes in the amino acids of the HVR1 allow HCV to escape humoral immunoglobulins and cause the infection to persist. Therefore analysis of genetic complexity of this region is imperative for understanding the role of envelope gene specifically HVR1 in virus interaction to target cells and its persistence which will effect the outcome of treatment. Rapid responders have recently been identified as positive predictors of sustained virological response in Pakistan [19]. Therefore our study was focused on the genetic complexity of HVR1 of envelope 2 protein in rapid responders in the pre-treatment samples with an aim of identifying some significant mutations or pattern of mutations associated specifically with the rapid responders. This study can therefore be helpful in predicting the outcome of treatment response even before the onset of treatment and tailoring the treatment regimen according to the predicted treatment response.

## Results

### Amino acids substitution analysis in HVR1

In HVR1, four amino acids position 2, 6, 19 and 23 were conserved in all the 31 variants. Conserved and variable sites among variants of rapid responders and among variants of breakthrough responders were also analyzed (Table-1 &2). Total number of conserved and variable sites was almost same in both groups but amino acid positions differed. Amino acid position 3, 7, 9 and 15 were conserved in breakthrough responders but had amino acid substitution in rapid responders. Conversely amino acids 13, 18, 24 and 26 were conserved in rapid responders but variable in breakthrough responders. Multiple amino acid substitution at single site was more common in rapid responders than in breakthrough responders for example at position 1 and 4 amino acids histidine (H) and threonine (T) were replaced by four other amino acids in rapid responders whereas in breakthrough responders at these positions single substitutions were observed.

**Table 1:** amino acids substitution in HVR1 of E2 protein (Rapid responder group)

*H*	T	*Y*	*T*	*T*	G	*G*	*T*	*A*	A	*R*	*N*	T	*K*	*G*	L	*A*	G	L	*F*	*Y*	*P*	G	A	*K*	Q	*K*
*1*	**2**	*3*	*4*	*5*	**6**	*7*	*8*	*9*	**10**	*11*	*12*	**13**	*14*	*15*	**16**	*17*	**18**	**19**	*20*	*21*	*22*	**23**	**24**	25	**26**	27
N5		Y8	T5	T10		G10	S7	V5		S5	S7		S10	G10		T7			F14	S10	R5			S10		N10
V4		H7	V5	V4		S5	K5	I5		R5	T5		R5	S5		V5			L1	T4	S5			K5		K3
Q2			I3	A1			N3	A5		H3	N2					A3				D1	P5					R1
H2			P1							Y2																W1
K1			L1																							

Amino acid substitution at each amino acid position analyzed from pre-treatment patient samples that belong to rapid responder group (R.R). R.R group (3 samples and 15 clones) contains 10 conserved sites and 17 variable sites

**Table 2:** amino acids substitution in HVR1 of E2 protein (Breakthrough responders)

*H*	T	Y	*T*	*T*	G	G	*T*	A	A	*R*	*N*	*T*	*K*	G	*L*	*A*	*G*	L	*F*	*Y*	*P*	G	*A*	*K*	*Q*	*K*
*1*	**2**	**3**	*4*	*5*	**6**	**7**	*8*	**9**	**10**	*11*	*12*	*13*	*14*	**15**	*16*	*17*	*18*	**19**	*20*	*21*	*22*	**23**	*24*	*25*	*26*	*27*
S9			T9	S9			S9			R9	D9	V8	H5		L9	V9	G9		F12	S14	P9		A9	Q9	Q12	R9
G5			V5	T5			Q5			S5	H5	T5	K9		F5	T5	N5		S		A5		P5	K5	R2	N5
												I1							L							

Amino acid substitution at each amino acid position analyzed from pre-treatment patient samples that belong to breakthrough responders (B.T). B.T group (2 samples and 14 clones) contains 9 conserved sites and 18 variable sites. Conserved sites are shown in bold and non-italic letters

Variable sites in rapid responders have some sites that maintained their physiochemical properties, these include amino acid 9 and 20 which were exclusively hydrophobic and non-polar amino. Another physiochemical stable site was of amino acid 21, which was exclusively polar neutral amino acid site, however one of the variant harbored polar acidic amino acid, aspartic acid (D) at amino acid position 21.

In breakthrough responders variable sites that maintained their physiochemical properties were amino acid 5 and 8 that were exclusively polar neutral sites. Amino acid 16 was exclusively non polar and hydrophobic, this amino acid is a conserved site in rapid responders as well. Amino acid 27 was also exclusively polar and hydrophilic in breakthrough responders, whereas in rapid responders this site was almost conserved with polar hydrophilic properties and very rare non polar amino acid substitution (1 out of 15 in our case).

Amino acid 16 and 27 maintained their physiochemical properties despite of amino acid substitution in breakthrough responders. Amino acid 16 was at conserved site while amino acid 27 maintained its physiochemical properties in rapid responders thus amino acid 16 and 27 can be considered as sites which are resistant to a functional change.

Similarly amino acids 9, 20 and 21 maintained their physiochemical properties in rapid responders were either at conserved sites in breakthrough responders or were resistant to physiochemical changes.

Five amino acids sites that were conserved sites in both rapid responders and breakthrough responders are 2, 6, 10, 19 and 23. Five sites that were either conserved sites in one of the two groups or that maintained their physiochemical properties in both the groups were 16, 27, 9, 20 and 21. In total ten amino acids sites were exclusively conserved between samples responding differently to the treatment. Remaining seventeen sites were displaying different physiochemical properties.

In rapid responders ten amino acid sites were conserved. Four amino acid sites were not conserved but maintained their physiochemical properties. Therefore among rapid responders more than half part of the HVR1 (fourteen amino acid sites) were stable functionally. In breakthrough responders nine amino acids were conserved. Amino acid substitution in four amino acid sites resisted any physiochemical change. Therefore like rapid responders almost half of the region of HVR1 remained stable functionally.

### Amino acid frequency

**Figure 1:**
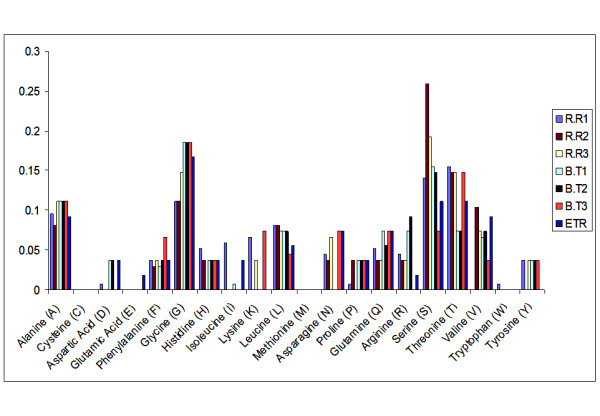
**Frequency of amino acids in the HVR 1 of samples responding differently to treatment**. Graphical representation of frequency of twenty amino acids in the HVR 1 of samples responding differently to treatment (rapid responders R.R, breakthrough responders B.T and sample showing response at the end of treatment E.T.R). Most frequently present amino acids were serine (S), Threonine (T), Glycine (G) and Alanine (A).

Frequency of individual amino acid in HVR1 was compared between samples (Figure 1). Some of the amino acids were equally frequent among all the samples. For example amino acid proline and tyrosine were equally frequent in all the samples but frequency of most of the amino acids was quite divergent between samples. Change in amino acids frequency was also analyzed within the sample (data not shown) i.e. at intrahost level since we had observed intrahost nucleotide variations. There were more amino acid substitutions among the variants of rapid responders and so there was divergent amino acid frequency among rapid responders but only in case of R1 sample. Amino acids alanine, glutamine, serine and threonine frequencies varied between variants of R1. However in case of other samples change in amino acid frequency was almost unnoticeable.

### Amino acid composition

Comparison between breakthrough and rapid responder group indicated that polar amino acids were slightly higher in rapid responder compare to breakthrough responder. In R.R group 56.73% of amino acids were polar and 43.28% of amino acids were non polar. In BT.R group 49.88% of amino acid were polar and 50.12% of amino acids were non polar. Among polar, neutral polar amino acids were more frequent than charged amino acids. Average composition of hydrophobic amino acids was greater than hydrophilic amino acids. Composition of hydrophilic amino acid was slightly greater in BT.R group (18.52%) than R.R group (17.6%). Acidic amino acids composition was slightly greater in breakthrough responders (2.22%) than in rapid responders (0.23%). (Tables-3 &4)

**Table 3:** Amino acid composition (polar and non polar amino acids) in HVR1 of E2 protein

**HVR 1**	**Polar a.a composition**			**Non polar a.a composition**
	
	**+ve charged (basic)**	**- ve charged(acidic)**	**Neutral**	
**RR group (15 variants)**	Lys(K)3.94	Asp(D).23	Ser(S)18.29	Ala(A)9.95
	His(H)3.01	Glu(E)-	Thr(T)15.28	Val(V)5.56
	Arg(R)3.94		Gln(Q)4.17	Leu(L)7.87
			Cys(C)-	Ile(I)1.85
			Asn(N)5.09	Gly(G)12.73
			Tyr(Y)2.78	Trp(W)0.23
				Phe(F)3.47
				Pro(P)1.62
				Met(M)-
**Average**	10.89	.23	45.61	43.28
**BT group (14 variants)**	Lys(K)3.21	Asp(D)2.22	Ser(S)11.60	Ala(A)11.36
	His(H)3.70	Glu(E)-	Thr(T)10.62	Val(V)5.43
	Arg(R)5.19		Gln(Q)6.67	Leu(L)6.42
			Cys(C)-	Ile(I).25
			Asn(N)2.72	Gly(G)18.52
			Tyr(Y) 3.95	Trp(W)-
				Phe(F)4.44
				Pro(P)3.70
				Met(M)-
**Average**	12.1	2.22	35.56	50.12

Comparison of average composition of twenty amino acids between rapid responders and breakthrough responders. Average composition of polar and non polar amino acids in HVR1 of rapid responders (R.R) and breakthrough responders (B.T).

**Table 4:** Amino acid composition (hydrophilic, hydrophobic and neutral amino acids) in HVR1 of E2 protein

	Hydropathic composition		
**HVR 1**	**Hydrophobic**	**Neutral**	**Hydrophillic**
**RR group (15 variants)**	Leu (L) 7.87	Thr (T) 15.28	Arg (R) 3.94
	Ile (I) 1.85	Glu (E)-	Lys (K)3.94
	Phe (F) 3.47	Gly (G) 12.73	Asn (N) 5.09
	Trp (W) .23	Ser (S) 18.29	His (H) 3.01
	Val (V) 5.56	Gln (Q) 4.17	Pro (P) 1.62
	Met (M) -	Asp (D) .23	
	Cys (C) -		
	Tyr (Y) 2.78		
	Ala (A) 9.95		
**Average**	31.71	50.7	17.6
**BT group (14 variants)**	Leu (L) 6.42	Thr (T) 10.62	Arg (R) 5.19
	Ile (I) .25	Glu (E) -	Lys (K)3.21
	Phe (F) 4.44	Gly (G) 18.52	Asn (N) 2.72
	Trp (W) -	Ser (S) 11.60	His (H) 3.70
	Val (V) 5.43	Gln (Q) 6.67	Pro (P) 3.70
	Met (M) -	Asp (D) 2.22	
	Cys (C) -		
	Tyr (Y) 3.95		
	Ala (A) 11.36		
**Average**	31.85	49.63	18.52

Comparison of average composition of twenty amino acids between rapid responders and breakthrough responders. Average composition of hydrophilic, hydrophobic and neutral amino acids in HVR1 rapid responders (R.R) and breakthrough responders (B.T)

### Analysis of antigenic profile

**Figure 2:**
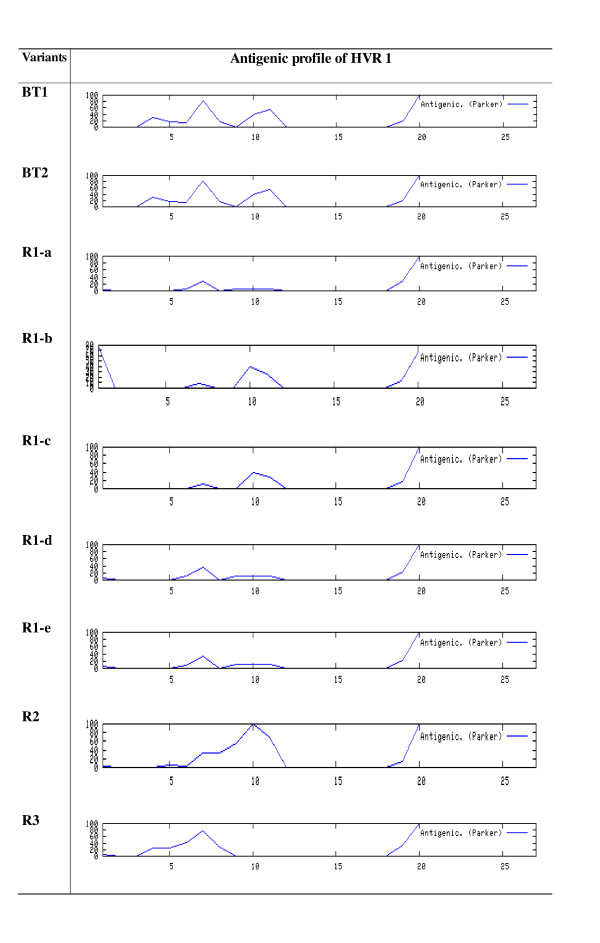
**Antigenic profile of HVR1**. Graphs comparing antigenicity computed by method of parker. (2) of HVR1 between breakthrough responders (BT1 BT2), rapid responders (R1 variants, R2 and R3) along HVR1 at each amino acid position.

All the variants supported the presence of two antigenic region separated by non antigenic region. Antigenic region towards the carboxyl terminal extended from amino acid 20-27 in all the samples and remained constant among variants. However the length of antigenic region towards the amino terminal varied from sample to sample. It extended from position 4-11 in breakthrough responders and remained constant in all the variants. However in rapid responders this antigenic region was quite variable. R1 was least antigenic with a slightly raised peak at position 6 and 10 (remember these amino acid positions were conserved). In R2 antigenic region extended from position 6 to 11 and remained constant in all variants of R2. In sample R3 antigenic region started a bit earlier and ended a bit earlier from position 4 to 8 (Figure 2).

## Discussion

Sequences of HVR1 of 31 variants from six samples consisting of 3 rapid responders (R1, R2 and R3) and two breakthrough responders (BT1 and BT2) were compared at amino acid level. Total number of conserved and variable sites remained almost same in both the groups opposing the common assumption that responders harbors less variability than the non responders. Fourteen out of twenty seven amino acid positions were resistant to any change in physiochemical properties in rapid responders. On the other hand half of the amino acids were conserved or resistant to physiochemical shifts in breakthrough responders. Although number of conserved and variable sites was same in both groups their positions varied between rapid responders and breakthrough responders. Ten amino acids sites were exclusively conserved between samples responding differently to the treatment. Half of these sites were either conserved in one of the two groups or retained their physiochemical properties. Other half of the amino acids did not harbor any substitution at all. Another study confirmed that despite of high variability in HVR1, at most of the position hydropathic characters remained conserved [17]. Another study proves the conservation of HVR 1 composition, length and amino acid substitution [20].

It has been already noted that neutral amino acids are more frequent in HVR1 than in common proteins [17]. It has been observed previously that neutral amino acids are more frequently present in HVR1 followed by hydrophobic and hydrophilic amino acids, our study supports the observation as neutral amino acids were found to be more frequent in HVR1 of both RR and BTR

Hydrophilic residues are exposed on the surface and are involved in interactions with other molecules whereas hydrophobic amino acids anchor E2 to core and maintains conformation of HVR1 [17]. In the present study composition of hydrophilic amino acids was slightly higher in breakthrough responders (18.52%) than in rapid responders (17.6%) whereas composition of hydrophobic amino acids was almost same in both the groups (31.71% in rapid responders and 31.85% in breakthrough responders). Among hydrophillic amino acids, those bearing basic properties were more common than those bearing acidic properties, confirming HVR1 to be a basic stretch as described previously by Penin, Combet et al. The basic nature of HVR1 made it to interact with lipids, GAG, s or proteins bearing negative charges [17,21]. Composition of basic residues was again slightly higher in breakthrough responders (12.1%) than rapid responders (10.8%). From above discussion we can conclude that changes in composition of hydrophilic and basic residues can lead to changes in the interaction with low density lipoprotein (LDL) and GAG's that are proposed to be involved in virus internalization [22] and receptor virus interaction [23] respectively thereby affecting infection cycle of HCV.

Most frequent amino acids in HVR1 were glycine (G). threonine (T), serine (S) and alanine (A) (Figure 1). These amino acids are small in size and flexible amino acids that can attain any conformation and their frequent presence balance the less flexible large amino acids [17]. However their frequency differed between samples as rapid responders had greater frequency of these amino acids (56.25%) than breakthrough responders (52.1%) making the earlier one more flexible. This ensures slight conformational variability between the two groups but these amino acids are either neutral or hydrophobic which means they would not be involved in interaction to other cells. Antigenic properties were also compared which revealed the presence of two antigenic domains as previously reported [17,24]. The antigenic domain towards the carboxyl terminal was stable however the one towards amino terminus was variable between samples. In other study antigenic characteristics at the amino terminus differed between lineages [25]. Our study confirmed the presence of a non antigenic domain in genotype 3a samples as reported in genotype 1b [24]. Results indicated that due to amino acid substitution at multiple sites antigenicity of HVR1 changed. Changes were quite significant among rapid responders than in breakthrough responders where antigenic region was quite constant in all variants that could be significant for the compatibility of HCV to the host immune response.

From our study and many other studies that stresses on the assumption that despite of uncontrolled hyper variability HVR1 maintains high conservation at many amino acid positions, and substitutions at variable sites do not maintain a specific pattern [26,27] We can conclude that although hyper variable region is quite variable region it still maintains its physiochemical properties which help it maintaining its structure ensures its viability and proper functioning. When comparing rapid responders with breakthrough responders we found slight differences as breakthrough responders were comparatively more likely to interact with target cells and cause infection.

Since this study points out only slight differences between rapid responders and breakthrough responders, further studies are still needed to be done on large number of samples to confirm the results and at the same time properties of other hyper variable regions in rapid responders are still needed to be studied.

## Conclusions

Hypervariable region is quite variable region in HCV 3a pretreatment samples responding differently to the treatment but it still maintains its physiochemical properties for proper functioning. Slight differences in rapid responders and breakthrough responders indicated that rapid responders were less likely to interact with target cells and cause infection. Antigenic variability was more significant in rapid responders than in breakthrough responders.

## Methods

### Patients and samples

Six patients infected with HCV 3a genotype were selected for this study. All of them were subjected to a combination therapy consisting of recombinant interferon (3 MU three times a week, subcutaneously) and ribavirin (10 mg/kg per day) for 6 months. Serum samples taken before the onset of treatment were examined. Three patients (R1, R2, and R3) showed a rapid response to treatment as defined by negative HCV RNA (<500 IU/ml) after 1 month of treatment. Two patients (BT1 and BT2) showed a breakthrough virological response during treatment followed by reappearance of HCV RNA at the end of treatment. One of the patient showed negative HCV RNA at the end of treatment and was defined as end of treatment response (ETR). The subjects gave informed consent to participate and the study was conducted in accordance with the 1964 Declaration of Helsinki and Guidelines for Good Clinical Research Practice in Pakistan. The study was approved by Ethics Committee of Molecular Virology Division.

### HCV RNA extraction and amplification of E2

Hepatitis C viral RNA was extracted from 100 μl of stored serum of the patients using Gentra RNA isolation Kit (Gentra, Purescript Technologies, USA) according to the kit protocol. cDNA synthesis of extracted RNA (10 μl) was done at 37°C for 50 min and 42°C for 10 min using 200 units of Moloney Murine Leukemia Virus reverse transcriptase (M-MLV RTase) (Invitrogen) and 8 Units of RNase inhibitor in a 20 μl of reaction with E2 outer anti sense primer (GTATTACGAGGTTCTCCAAAGC). For nested PCR amplification of entire E2 gene two sets of primers were optimized. The first round of PCR was done with 10 pM of E2 outer antisense primer and with 10 pM of outer sense primer (TCCTGGTAGTGCTGCTGCTA). Four microliter of the first round PCR products was used as a template for second round of nested PCR using inner sense (GAAACCCACGTCACCGGGGGAA) and inner antisense primers (CGCCTCCGCTTGGGATATGAGTAACA). The amplification conditions were same for both PCR rounds: initial denaturation at 94°C for 2 min followed by 35 cycles consisting of denaturation at 94°C for 45 sec, primer annealing at 50°C for 45 sec and primer extension at 72°C for 3 min followed by a final extension of 10 min. The amplified products were visualized on 1.2% agarose gel stained with ethidium bromide under UV transilluminator after electrophresis. The required bands were excised and DNA was purified from the agarose gel with Silica Bead DNA Gel Extraction Kit (Fermentas Inc. Germany). Purified DNA was suspended in depc treated water and used for cloning and sequencing.

### Cloning of E2

Purified E2 amplified products were directly ligated to 50 ng of PCR 2.1 vector (TA Cloning Kit; Invitrogen). The product was transformed into chemically competent TOP10F' cells by incubating the entire ligation reaction along with competent cells on ice for 30 min then a 1 minute heat shock was given to cells at 42°C after which the cells were immediately transferred to ice. Then the cells were incubated with 500 ul of Luria-Bertani (LB) medium at 37°C for 1 hour. Successfully transformed cells were selected on LB agar plate supplemented with 100 μg/ml ampicillin and 12.4 ug/ml tetracycline. Plates were subjected to overnight incubation at 37°C. Clones carrying E2 gene were confirmed by colony PCR. 50 ul of culture was centrifuged at 13000 rpm for 2 min. supernatant was discarded and pellet was resuspended in 50 μl of 1X T.E. resuspended pellet was subjected to heat shock at 100°C for 10 min followed by centrifugation at 13000 rpm for 2 min. Supernatant that was supposed to contain the plasmid was used as a template for the PCR reactions. These PCR reactions were prepared both with 10 pmol vector-specific primers M13 forward (TAATACGACTCACTATAGGG) and M13 reverse (TAGAAGGCACAGTCGAGG) and with gene specific primers (E2 inner sense and inner antisense). Bands of size approximately 1056 bp with gene specific primers and 1183 with vector specific primers were visualized under UV confirmed successful cloning. Plasmid was isolated from cultures containing E2 clones using a Plasmid Miniprep Kit, (Fermentas Life Science technologies USA) according to manufacturer's protocol. From each patient 5-10 positive clones were randomly selected for sequencing.

### Sequencing

For each clone, both strands of E2 gene were sequenced with M13 forward and reverse primers by Applied biosystems prism dye termination method according to the manufacturer's instructions (Big Dye Deoxy Terminators; Applied Biosystems, Weiterstadt, Germany). Sequencing was performed on automated sequencer (Applied Biosystems; 3100 DNA Analyzer). Sequencing PCR reaction was performed with profile; 96°C for 15 sec, 50°C for 10 sec, 60°C for 4 min repeated 35 times. Labeled DNA was then purified with 2 μl of 3M Sodium Acetate and 2 μl of 125 mM EDTA followed by precipitation with 26 μl 100% ethanol. After 15 min incubation at room temperature DNA was centrifuged for 30 minutes at 2800 rpm at 4°C. Washing was done with 36 μl of 70% ethanol. Pellet was air dried and rehydrated in 12 μl formamide. Pellet was chilled on ice after it was heat shocked at 95°C for 5 minutes. Air dried pellet was then analyzed using an ABI prism sequencer. A set of 31 variants was analyzed containing 2-10 clones selected from each patient. (5 clones from patient R1, R2, R3, BT2; 9 clones from patient BT1 and 2 clones from patient ETR). Sequences were analyzed with the Chromas LITE programme (version 2.01; Technelysium Pty Ltd).

### Amino acid heterogeneity analysis of HVR1

Amino acid heterogeneity analysis was done by aligning amino acids using Bioedit clustal W programme. These aligned sequences were then analyzed using MEGA version 4.1 [28]. Number of conserved sites, number of variable sites, amino acid substitution at each amino acid position (1-27 a.a) and amino acid composition was calculated for each variant and then comparisons were made between samples, between variants and groups. Amino acid frequency, count of hydrophilic and hydrophobic residues and count of charged and uncharged residues were done by CLC workbench software http://www.clcbio.com.

### Antigenic profile

Antigenic regions were identified within the HVR1 of Envelope protein by analyzing antigenic profiles of all the variants by the method of Parker et al. [29].

### Nucleotide sequence accession numbers

Accession numbers of nucleotide sequences submitted to GenBank are HM590012 to HM590017, HM584119 to HM584121, HM462252, HQ184929 to HQ184935, HQ202715 to HQ202721 and HQ189124 to HQ189130

## Competing interests

The authors declare that they have no competing interests.

## Authors' contributions

MI conceived the study, participated in its design and coordination and gave a critical view of manuscript writing. MA performed and analyzed the results. SA, AH, MI, MA, SB and BK participated in results analysis. All the authors read and approved the final manuscript.
